# Early Father Involvement and Subsequent Child Behaviour at Ages 3, 5 and 7 Years: Prospective Analysis of the UK Millennium Cohort Study

**DOI:** 10.1371/journal.pone.0162339

**Published:** 2016-09-21

**Authors:** Mary E. Kroll, Claire Carson, Maggie Redshaw, Maria A. Quigley

**Affiliations:** Policy Research Unit in Maternal Health and Care, National Perinatal Epidemiology Unit, Nuffield Department of Population Health, University of Oxford, Oxford, United Kingdom; University Children's Hospital Tuebingen, GERMANY

## Abstract

**Objective:**

Fathers are increasingly involved in care of their babies and young children. We assessed the association of resident fathers’ involvement with subsequent behaviour of their children, examining boys and girls separately.

**Methods:**

We used longitudinal data from the UK Millennium Cohort Study for children born in 2000–2001, divided into three separate analysis periods: ages 9 months to 3 years, 3 to 5 years, and 5 to 7 years. By exploratory factor analysis of self-reported attitudes and engagement in caring activities, we derived composite measures of various types of father involvement at 9 months, 3 and 5 years. Where possible we created equivalent measures of mother involvement. Child behaviour was assessed by the Strengths and Difficulties Questionnaire (SDQ), which was completed by the mother when the child was aged 3, 5 and 7 years. We estimated gender-specific odds ratios for behaviour problems per quintile of father involvement, using separate logistic regression models for boys and girls in each analysis period. We controlled for a wide range of potential confounders: characteristics of the child (temperament and development at 9 months, and illness and exact age at outcome), equivalent mother involvement where appropriate, and factors related to socioeconomic status, household change, and parental well-being, where statistically significant.

**Results:**

Paternal positive parenting beliefs at age 9 months and increased frequency of creative play at age 5 years were significantly associated with lower risk of subsequent behaviour problems (SDQ total difficulties) in both boys and girls (p<0.05), odds ratios ranging between 0.81 and 0.89 per quintile of involvement. No associations were observed for other composite measures of caring activity by the father at 9 months, 3 years or 5 years.

**Conclusion:**

Quality of parenting, rather than the division of routine care between parents, was associated with child behavioural outcomes.

## Introduction

Involvement of UK fathers in the care of their infants and young children increased dramatically during the second half of the twentieth century, both in total time spent and as a proportion of all parental childcare [1]. Between 2002 and 2005, with the introduction of statutory paternity leave and pay in 2003, the proportion of UK fathers taking more than two weeks leave around childbirth increased from 22% to 36%, and the proportion of UK fathers with access to flexi-time increased from 22% to 54% [2]. Since April 2015 two parents have been allowed to share the UK statutory parental leave entitlement (currently up to 52 weeks, with pay for up to 39 weeks) [3]. Similar changes are under way in many other countries [4]. Nevertheless, support for new parents in the UK still tends to focus on mothers, and to be more actively used by women, and it has been suggested that greater efforts to include fathers might improve long-term outcomes for children [5].

The standard family environment model [6] predicts that positive father involvement might aid the development of emotional regulation, social skills, and other aspects of child behaviour [7]. Many areas of psychological functioning appear to be influenced by parental characteristics such as maternal or paternal psychopathology [8, 9], absence [10], partner relationship [11] and ethnicity [12]. Closeness to parents in childhood may lead to good relationships with parents and partners in adolescence and adult life [13].

Existing evidence for direct specific benefits of father involvement to child behaviour is limited, however [14]. Definitions of ‘father involvement’ have varied over time, and tended to focus on co-residence and frequency of contact [15]. A recent review has argued that there may be no real difference between the influence of the mother and the father, and suggested that future researchers should include both parents in their models, use the same measures of involvement for each parent where possible, and consider quality (e.g. positive engagement, warmth, responsiveness) as well as quantity of parenting [16]. Effects might be different for boys and girls, as there are well-established gender differences in normal behaviour, which may in part be biological [17].

We aimed to clarify the relationship of early father involvement to child behavioural outcomes for two-parent families in the UK, using recent national cohort data. We assessed various forms of father involvement, controlled for equivalent mother involvement where available, and analysed data on boys and girls separately.

## Materials and Methods

### Millennium Cohort Study

The UK Millennium Cohort Study (MCS) follows a nationally-representative cohort of children born around 2001 [18]. Electoral wards were selected at random from each of the four countries of the UK. Children were eligible for the cohort if they were living in a sampled electoral ward at age 9 months according to records of eligibility for Child Benefit (a nearly-universal social security payment), and were born from 1 September 2000 to 31 August 2001 (for England and Wales), or from 24 November 2000 to 11 January 2002 (for Scotland and Northern Ireland) [18]. Wards with high levels of child poverty and/or high proportions of children from ethnic minorities were over-sampled to improve reliability of inference for these important subpopulations; to compensate for disproportionate stratification, sample design weights are provided with the data [18], and we used these in our analysis. Interview questions were devised by a team based at the Centre for Longitudinal Studies (Institute of Education, London University) in consultation with potential users and collaborators [18]. Members of participating families were interviewed at home by trained research staff when the children were aged approximately 9 months (18,552 families) [19], 3 years [20], 5 years [21] and 7 years [22].

### Study populations

We divided the study into three separate analysis periods (exposure to outcome) defined by the availability of MCS interview data: ages 9 months to 3 years, 3 to 5 years, and 5 to 7 years. All analyses were restricted to participating families with term (37+ weeks) singleton births (16,809 families at 9 months). Of these, respectively 80%, 71% and 69% (13,503, 11,995 and 11,570 families) participated at both exposure and outcome in the three analysis periods; to compensate for possible participation bias, weights based on the characteristics of non-responders are provided with the data for each survey [18], and we used these in our analyses. Further restrictions were as follows: at exposure, both birth parents were resident full-time, the mother gave the ‘main carer’ interview, and the father gave the ‘partner’ interview; at outcome, only English was spoken in the household, and the mother gave the ‘main carer’ interview. These criteria yielded study populations of 8408, 6970 and 6747 children respectively in the three analysis periods.

### Child behaviour at outcome

In the MCS, child behaviour was assessed by a pre-existing standard psychological measure, the Strengths and Difficulties Questionnaire (SDQ) [23], which was completed by the ‘main carer’ (i.e. the mother, in our study populations) at ages 3, 5 and 7 years. Parent-reported SDQ is widely used in research and has been validated for children aged 3 to 12 years [24–26]. We used two mutually exclusive summary SDQ scales: ‘total difficulties’ and ‘prosocial strengths’. The former is the sum of subscales for four different types of behavioural difficulties: emotion, conduct, attention and peer problems. The latter is the sum of scores for five positive characteristics, i.e. ‘considerate of other people's feelings’, ‘shares readily with other children’, ‘helpful if someone is hurt’, ‘kind to younger children’, ‘often volunteers to help others’; similar measures of prosocial behaviour have been found to predict better social adjustment independently of co-existing disruptive or aggressive behaviour [27, 28]. We considered the child to have potential for ‘behaviour problems’ if the SDQ score was in the extreme 10% of the distribution of scores over the whole MCS cohort at ages 3, 5 and 7 years [29], i.e. the top decile for the total difficulties scale (17+, 14+, 15+) and the bottom decile for the prosocial scale (< = 5, < = 6, < = 6) respectively.

### Parent involvement at exposure

From the available MCS interview data for each analysis period, we selected variables that described father involvement, e.g. strength of agreement with ‘positive’ parenting beliefs, frequency of caring activities, and division of these activities with the mother (Table 1). We scored the variables as 0,1,2,…, a higher score indicating greater involvement (Table 2), and grouped them by exploratory factor analysis, using the ‘principal factors’ method with varimax rotation [30]. We selected factors using eigenvalue scree plots, and chose a factor-loading threshold of 0.3, taking the higher-loaded variable where there was cross-loading (Table 1). Sampling adequacy was acceptable (Kaiser-Meyer-Olkin measure >0.6), and the resulting factors showed acceptable reliability/consistency (Cronbach’s alpha >0.5). Single variables that did not load on any factor and did not vary enough to be informative on their own were dropped.

**Table 1:** Father involvement variables: rotated loadings from exploratory factor analysis.

Father involvement variables, by age of child at survey	Factor loadings, where >0.3		
**Age 9 months**	**Sharing care**	**Feeding etc.**	**Beliefs**
Have you taken any leave from any job to be at home with the baby?	-	-	-
Who has most responsibility for feeding the baby? (reported by mother)	0.86	-	-
Who has most responsibility for changing nappies? (reported by mother)	0.85	-	-
Who does most looking after children generally? (reported by mother)	0.75	-	-
How often do you feed the baby?	(0.40)	0.67	-
How often do you change the baby’s nappy?	(0.39)	0.69	-
How often do you look after the baby on your own?	-	0.43	-
It is important to develop a regular pattern of feeding and sleeping with a baby	-	-	0.45
Babies need to be stimulated if they are to develop well	-	-	0.72
Talking, even to a young baby, is important	-	-	0.83
Cuddling a baby is very important	-	-	0.70
Children need father to be as closely involved in their upbringing as mother	-	-	0.44
**Age 3 years**	**Bedtime etc.**	**N/A**	**N/A**
Children need father to be as closely involved in their upbringing as mother	-	-	-
How often do you get the child ready for bed or put the child to bed?	0.48	-	-
How often do you read to the child?	0.44	-	-
How often do you play with the child?	0.38	-	-
**Age 5 years** How often do you…	**Bedtime etc.**	**Active play**	**Creative**
get the child ready for bed or put the child to bed?	0.51	-	-
read to the child?	0.48	-	-
look after the child on your own?	-	-	-
play with toys or games indoors with the child?	-	0.53	(0.33)
play sports or physically active games outdoors or indoors with the child?	-	0.58	-
take the child to the park or to an outdoor playground?	-	0.44	-
draw, paint or make things with the child?	-	(0.33)	0.48
listen to or play music, sing, dance, or do other musical activities with the child?	-	-	0.45
tell stories to the child not from a book?	-	-	0.46

Brackets show cross-loadings. MCS interview questions. Respondent was the father unless stated otherwise.

**Table 2:** Distributions of parent involvement variables.

Weighted %	P	BOY							GIRL						
Involvement score		0	1	2	3	4	5	-	0	1	2	3	4	5	-
**FATHER**															
**Sharing at 9 mth**															
Feeding (A)	0.7	76.6	20.6	0.8	-	-	-	2.0	77.4	19.6	0.9	-	-	-	2.1
Nappies (A)	0.2	67.8	29.5	1.2	-	-	-	1.6	69.6	27.5	1.0	-	-	-	1.9
Looking after (A)	0.4	62.0	36.1	0.9	-	-	-	1.0	62.6	35.3	0.7	-	-	-	1.4
**Feeding etc. at 9mth**															
Feeding (B)	0.1	2.5	4.0	10.8	28.1	28.6	26.0	0.0	2.3	4.5	10.9	30.3	28.8	23.2	0.0
Nappies (B)	0.3	3.3	5.9	7.6	22.7	21.2	39.2	0.0	4.2	6.1	7.5	24.2	20.7	37.3	0.0
Looking after (B)	0.1	2.7	11.4	25.9	29.4	13.8	16.7	0.0	2.1	11.8	28.0	28.8	14.2	15.1	0.1
**Positive beliefs at 9mth**															
Regularity (C)	0.7	0.2	1.0	3.6	34.0	59.6	-	1.6	0.1	1.2	4.0	34.1	59.0	-	1.6
Stimulation (C)	0.9	0.0	0.1	2.7	24.8	70.4	-	2.0	0.1	0.1	2.5	25.5	70.2	-	1.7
Talking (C)	0.8	0.1	0.0	0.5	15.7	82.5	-	1.2	0.0	0.0	0.4	16.1	82.2	-	1.3
Cuddling (C)	0.7	0.0	0.1	1.3	22.3	75.1	-	1.2	0.0	0.1	1.1	21.6	75.8	-	1.3
Fathering (C)	0.2	0.1	1.2	4.1	38.9	54.2	-	1.5	0.1	1.5	4.2	41.0	51.9	-	1.4
**Bedtime etc. at 3yr**															
Bedtime (B)	<0.001	3.1	6.3	15.5	49.9	24.1	1.0	0.0	3.8	6.7	19.4	48.8	20.7	0.6	0.0
Reading (D)	0.6	4.2	4.3	10.1	32.3	26.5	22.5	0.0	3.9	4.3	9.1	31.2	28.3	23.2	0.0
Play (B)	<0.001	0.1	0.6	3.2	15.6	36.5	44.1	0.0	0.1	0.4	5.3	17.4	36.9	39.9	0.0
**Bedtime etc. at 5yr**															
Bedtime (D)	0.003	2.1	3.0	5.0	21.0	48.2	20.7	0.0	2.7	3.8	4.9	23.6	48.1	16.7	0.2
Reading (D)	0.8	2.6	4.7	10.2	32.8	34.7	15.0	0.0	2.8	3.9	10.6	33.1	34.1	15.4	0.1
**Looking after at 5yr**															
Looking after (D)	<0.001	1.5	5.8	18.7	37.0	29.4	7.7	0.0	1.7	8.7	20.3	37.4	25.2	6.7	0.1
**Active play at 5yr**															
Indoor toys (D)	<0.001	0.6	2.6	6.7	33.2	38.5	18.4	0.0	1.0	5.1	11.3	38.6	31.0	12.9	0.1
Sports etc. (D)	<0.001	1.4	4.0	11.1	36.6	31.0	16.0	0.1	2.6	7.1	17.0	42.1	22.6	8.6	0.1
Playground (D)	<0.001	2.4	12.1	33.7	41.4	8.9	1.5	0.1	3.7	13.2	39.3	36.1	6.8	0.8	0.1
**Creative play at 5yr**															
Art/making (D)	0.06	5.8	16.7	29.9	34.7	10.2	2.6	0.0	6.0	17.2	30.0	31.5	12.2	3.1	0.1
Music/dance (D)	<0.001	7.0	11.0	15.2	27.4	22.9	16.5	0.0	4.7	8.6	13.5	28.7	25.6	18.8	0.1
Story-telling (D)	0.6	11.3	18.3	19.5	26.7	15.4	8.8	0.0	11.9	18.2	20.6	25.2	15.8	8.1	0.1
**MOTHER**															
**Positive beliefs at 9mth**															
Regularity (C)	0.7	0.6	1.4	6.0	38.7	52.6	-	0.6	0.5	1.6	6.5	39.3	51.5	-	0.7
Stimulation (C)	0.8	0.2	0.3	2.7	29.4	66.6	-	0.8	0.3	0.3	2.4	28.8	67.3	-	0.8
Talking (C)	0.7	0.1	0.0	0.1	14.5	84.8	-	0.5	0.2	0.0	0.2	15.1	84.0	-	0.5
Cuddling (C)	1.0	0.1	0.0	0.5	13.5	85.2	-	0.5	0.2	0.0	0.5	13.8	84.9	-	0.5
Fathering (C)	0.7	0.1	1.5	6.6	42.9	48.0	-	0.8	0.3	1.6	7.2	42.3	47.8	-	0.9
**Active play at 5yr**															
Indoor toys (D)	0.2	1.2	4.0	8.6	32.4	33.3	20.6	0.0	1.1	3.0	9.6	33.9	31.6	20.9	0.0
Sports etc. (D)	<0.001	6.0	13.4	18.1	36.8	19.2	6.6	0.0	5.8	13.6	22.0	38.8	15.6	4.3	0.0
Playground (D)	0.6	2.5	7.6	30.3	42.7	14.0	2.8	0.0	3.0	7.8	31.5	42.4	12.9	2.4	0.1
**Creative play at 5yr**															
Art/making (D)	<0.001	2.5	9.6	28.2	36.8	17.7	5.1	0.0	2.3	7.0	22.0	38.6	21.3	8.9	0.0
Music/dance (D)	<0.001	2.0	4.7	9.8	23.2	27.5	32.8	0.0	1.0	1.9	6.7	22.0	31.0	37.5	0.0
Story-telling (D)	0.2	11.4	18.9	18.4	25.4	16.0	9.8	0.1	10.1	16.9	18.9	26.9	16.4	10.8	0.0

Weighted % of study population for analysis period. See Table 1 for wording of MCS interview questions. Involvement score: 0 = low, … 5 = high. (A) mother does most, equal, father does most; (B) never, <1/wk, 1-2/wk, 3-6/wk, 1/day, >1/day; (C) strongly disagree, disagree, neither, agree, strongly agree; (D) never, <1/mth, 1-2/mth, 1-2/wk, 3-6/wk, 1+/day. P, p-value from χ^2^ test for difference between boys and girls.

**Fig 1:**
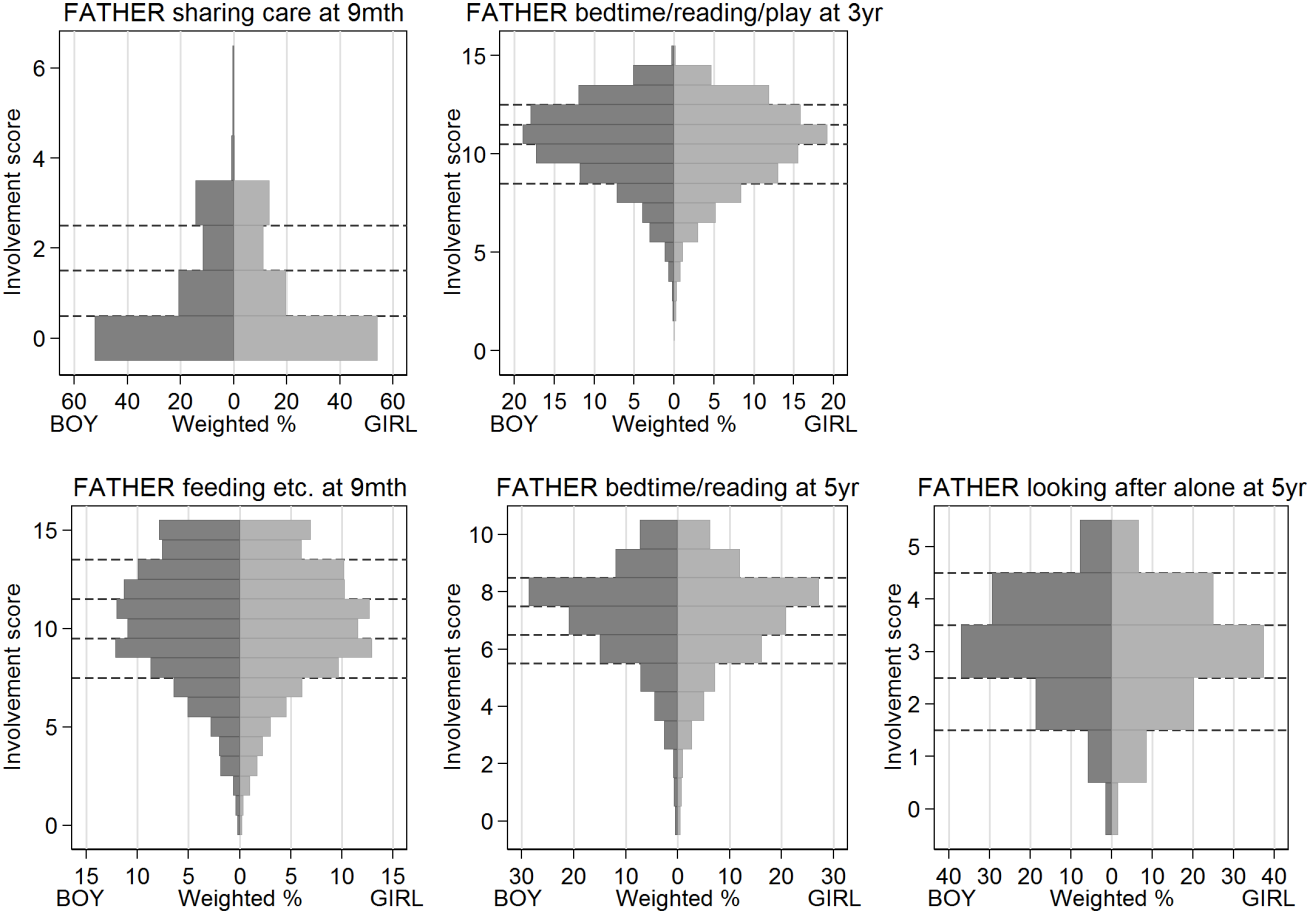
Distributions of father-only composite involvement measures. Weighted % of study population for analysis period, excluding missing values. Dashed lines show categories used in regression analysis.

**Fig 2:**
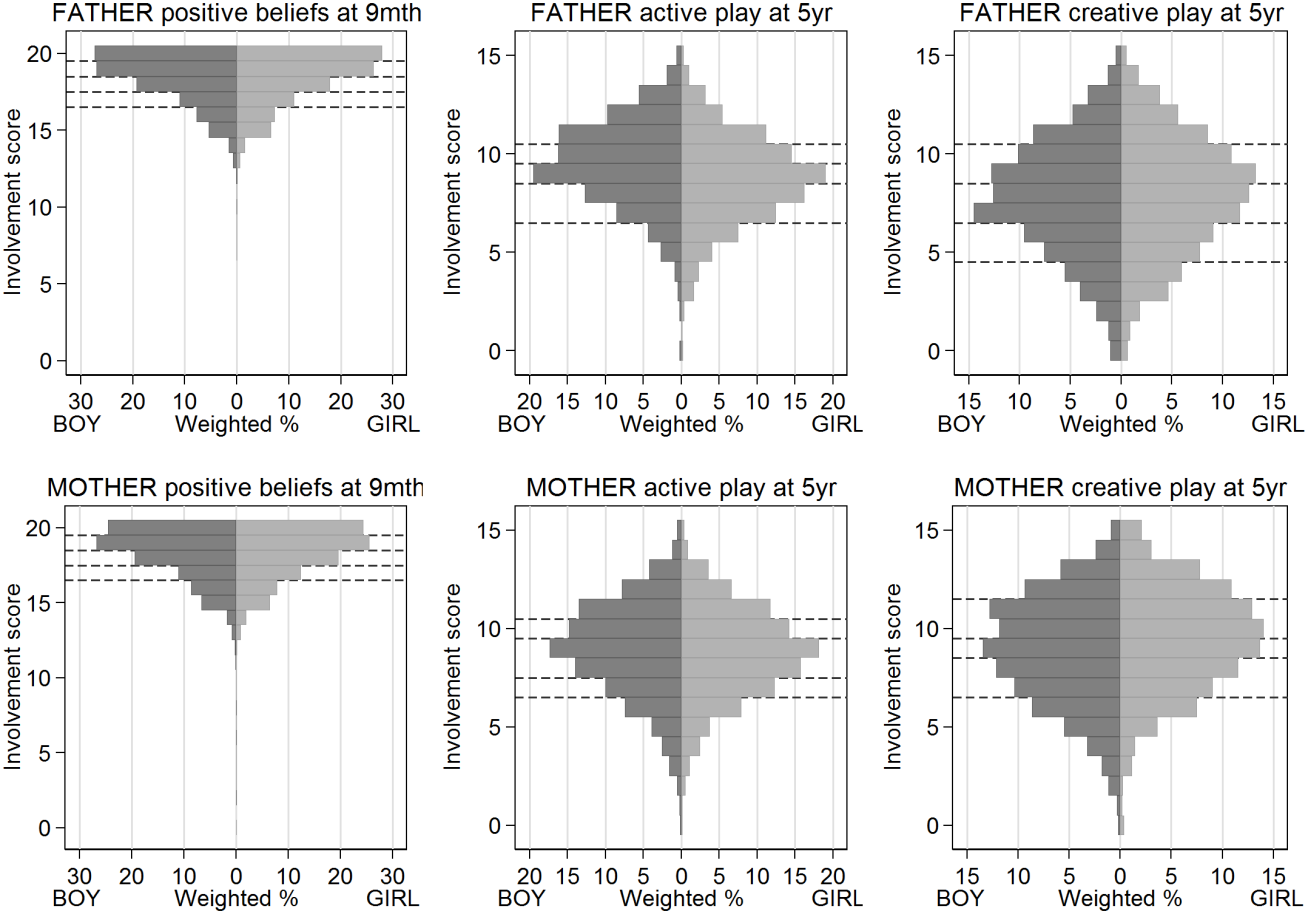
Distributions of both-parent composite involvement measures. Weighted % of study population for analysis period, excluding missing values. Dashed lines show categories used in regression analysis.

For each latent factor identified by the factor analysis (Table 1), we created a composite measure of father involvement by adding up the scores for the individual variables within the group (Table 2) [31]. Where exactly equivalent data were available, we created equivalent composites for mothers, using the same component variables and scoring system as for fathers (Table 2). Five composites were available only for fathers (Fig 1), and three were available for both parents (Fig 2). For analysis, we split each composite into approximate quintile categories.

### Statistical analysis

Separately for boys and girls, we fitted logistic regression models for risk of behaviour problems assessed by each SDQ scale (total difficulties and prosocial) in each analysis period [30]. The primary measure of association was the adjusted odds ratio for behaviour problems per quintile of parent involvement. The assumption of a linear trend over categories of involvement was checked using a Wald test. We used complete case analysis because the proportion of missing values in the study population was small (5% or less) for each regression variable. Distributions of involvement measures were compared using χ^2^ tests. All analyses were weighted to allow for the disproportionately-stratified cluster design of the cohort, and for non-participation at the relevant surveys. All statistical tests used the 5% significance level.

Except for the child’s age, all potential confounders were categorical factors derived from parental interview data. All adjusted models included four child factors *a priori*: at 9 months, temperament (approximate quintiles of a continuous measure based on items from the Carey infant temperament scale [32], including the 8% of children with incomplete data as an extra category) and any developmental delay (no, yes; based on items from the Denver [33] and McArthur [34] inventories); at outcome, exact age in days (continuous) and any long-standing limiting illness (no, yes). Where available, we also adjusted for the equivalent co-parent involvement measure (e.g. mother’s parenting beliefs if the exposure of interest was the father’s parenting beliefs).

Other potential confounders were added to the model in three stages, dropping any new additions that did not make a statistically significant contribution to the fit of the model at each stage, and any that did not remain statistically significant at the end of the modelling process (Table 3). (I) Factors related to socioeconomic status: mother’s (<25, 25–29, 30–34, 35+) and father’s (<30, 30–34, 35+) age in years at birth of child; mother’s and father’s education at 9 months (National Vocational Qualification equivalent level 4–5 = university degree or equivalent, 3 = A levels or equivalent, 2 = grade C or higher in at least 4 General Certificate of Secondary Education qualifications at the end of compulsory schooling at age 16, 0–1 = less than this); occupational socioeconomic status at exposure, based on the last-known job of father or mother, whichever was higher (3-class National Statistics Socio-economic Classification 1 = managerial/administrative/professional, 2 = intermediate, 3 = routine/manual); duration of breast-feeding (never, <4 months, 4+ months); number of siblings of child in household at 9 months (none, 1, 2+). (II) Household changes during analysis period: change in number of resident siblings (no, yes); absence of birth father at outcome (no, yes). (III) Parental well-being at exposure: mother’s long-standing limiting illness (no, yes); father’s work hours (none, <38, 38-<43, 43-<50, 50+) per week including overtime; mother’s and father’s symptoms of depression (no, yes) using the 9-item Rutter Malaise Inventory (4+) at 9 months [35] and 6-item Kessler Psychological Distress Scale (9+) at 3 and 5 years [36].

**Table 3:** Confounders included in adjusted statistical models.

Analysis period	9 months—3 years						3–5 years		5–7 years							
Parent involvement	Sharing		Feeding		Beliefs		Bedtime		Bedtime		Look after		Active		Creative	
	Boy	Girl	Boy	Girl	Boy	Girl	Boy	Girl	Boy	Girl	Boy	Girl	Boy	Girl	Boy	Girl
**Socioeconomic factors**																
Mother's age	T	**-**	T	T	T	T	**-**	**-**	T	P	**-**	**-**	T	P	T	**-**
Father's age	T	**-**	T	**-**	T	**-**	**-**	**-**	**-**	**-**	**-**	**-**	**-**	**-**	**-**	**-**
Mother's education	T	T	T	T	T	T	T	**-**	**-**	**-**	T	**-**	T	**-**	**-**	**-**
Father's education	**-**	P	**-**	P	**-**	P	**-**	**-**	**-**	T	**-**	T	**-**	T	**-**	T
Occupational status	T	T	T	T	**-**	T	T	T	**-**	**-**	**-**	**-**	**-**	**-**	**-**	**-**
Breastfeeding duration	T	T	T	**-**	T	**-**	T	T	T	T	**-**	T	**-**	T	**-**	T
No of siblings at 9mth	P	P	P	P	P	P	P	**-**	T	**-**	**-**	**-**	**-**	**-**	**-**	**-**
**Household changes**																
Number of siblings	T	**-**	T	**-**	T	**-**	T	P	T	**-**	T	**-**	T	**-**	T	**-**
Presence of father	**-**	T	**-**	**-**	**-**	**-**	**-**	T	**-**	TP	**-**	T	**-**	TP	**-**	TP
**Parental well-being**																
Mother's illness	T	**-**	T	**-**	T	**-**	**-**	**-**	T	T	T	T	T	T	T	T
Father’s work hours	**-**	**-**	**-**	**-**	**-**	**-**	**-**	**-**	**-**	**-**	**-**	**-**	**-**	**-**	**-**	**-**
Mother's depression	T	T	T	T	T	T	TP	T	T	TP	T	TP	T	TP	T	TP
Father's depression	**-**	**-**	**-**	**-**	**-**	**-**	**-**	**-**	T	**-**	T	**-**	T	**-**	T	**-**

Child behaviour measure: T = SDQ total difficulties, P = SDQ prosocial. All adjusted models also included child’s temperament and developmental delay at 9 months, child’s age and long-standing limiting illness at outcome, and (where available) co-parent involvement at exposure. See Tables 1 and 2 for details of parent involvement variables.

### Ethical approval

Our study was a secondary analysis of MCS datasets that had been deposited in the UK Data Archive. Ethical approval to conduct the original study was granted by the South West (MREC/01/6/19), London (MREC/03/2/022), London (05/MRE02/46) and Northern/Yorkshire (07/MRE03/32) Multi-Centre Medical Research Ethics Committees.

## Results

### Characteristics of the study populations

Low occupational status, parental depression, change in the number of siblings in the household (e.g. due to birth of a new sibling), and absence of the birth father at outcome (e.g. due to family breakdown) were more frequent in the study population for the first analysis period (Table 4). Longstanding limiting illness in child and/or mother was more frequent in later periods. Other characteristics were similar over the three periods.

**Table 4:** Characteristics of study populations.

Analysis period		9mth-3yr	3-5yr	5-7yr
Total number of children		8408	6970	6747
**Child**	Exact age at outcome, mean years	3.12	5.21	7.22
Girl, %		49.0	49.6	49.3
Never breast-fed, %		26.3	25.3	28.0
Developmental delay at 9 months, %		13.1	12.8	13.3
Temperament problems at 9 months, %		12.4	12.3	12.7
Long-standing limiting illness at outcome, %		2.5	4.6	5.3
**Father**	Age at birth of child, mean years	32	32	32
Low educational attainment (NVQ equivalent level 1 or less) at 9 months, %		15.6	15.1	15.3
Not working at exposure, %		7.0	6.0	6.5
Symptoms of depression at exposure, %		8.3	5.9	6.2
**Mother**	Age at birth of child, mean years	30	30	30
Low educational attainment (NVQ equivalent level 1 or less) at 9 months, %		13.6	13.3	14.2
Long-standing limiting illness at exposure, %		8.2	7.7	12.1
Symptoms of depression at exposure, %		11.3	6.8	6.9
**Household**				
Child had 2+ siblings in household at 9 months, %		20.2	19.9	20.4
Low occupational socioeconomic status (3-class NS-SEC 3) at exposure, %		22.0	19.3	18.9
Number of siblings in household changed during period, %		29.6	20.9	13.1
Birth father not resident at outcome, %		6.8	6.3	5.5

Weighted % of combined study population of boys and girls, excluding missing values. Full-term singleton births in families where the birth father was resident and interviewed at exposure, the family was English-speaking at outcome, and the birth mother gave the ‘main carer’ interview at exposure and outcome.

### Parent involvement variables

Some of the individual parent involvement variables differed by the sex of the child (Table 2). At age 3 years, the father more often played with the child or got the child ready for bed if the child was a boy. At age 5 years, the father more often looked after the child alone or engaged in any kind of active play if the child was a boy, but more often engaged in musical activities if the child was a girl. Similarly, at age 5 years, the mother more often engaged in physically active play if the child was a boy, and in artistic and musical activities if the child was a girl.

### Composite involvement measures that were available only for fathers

**Fig 3:**
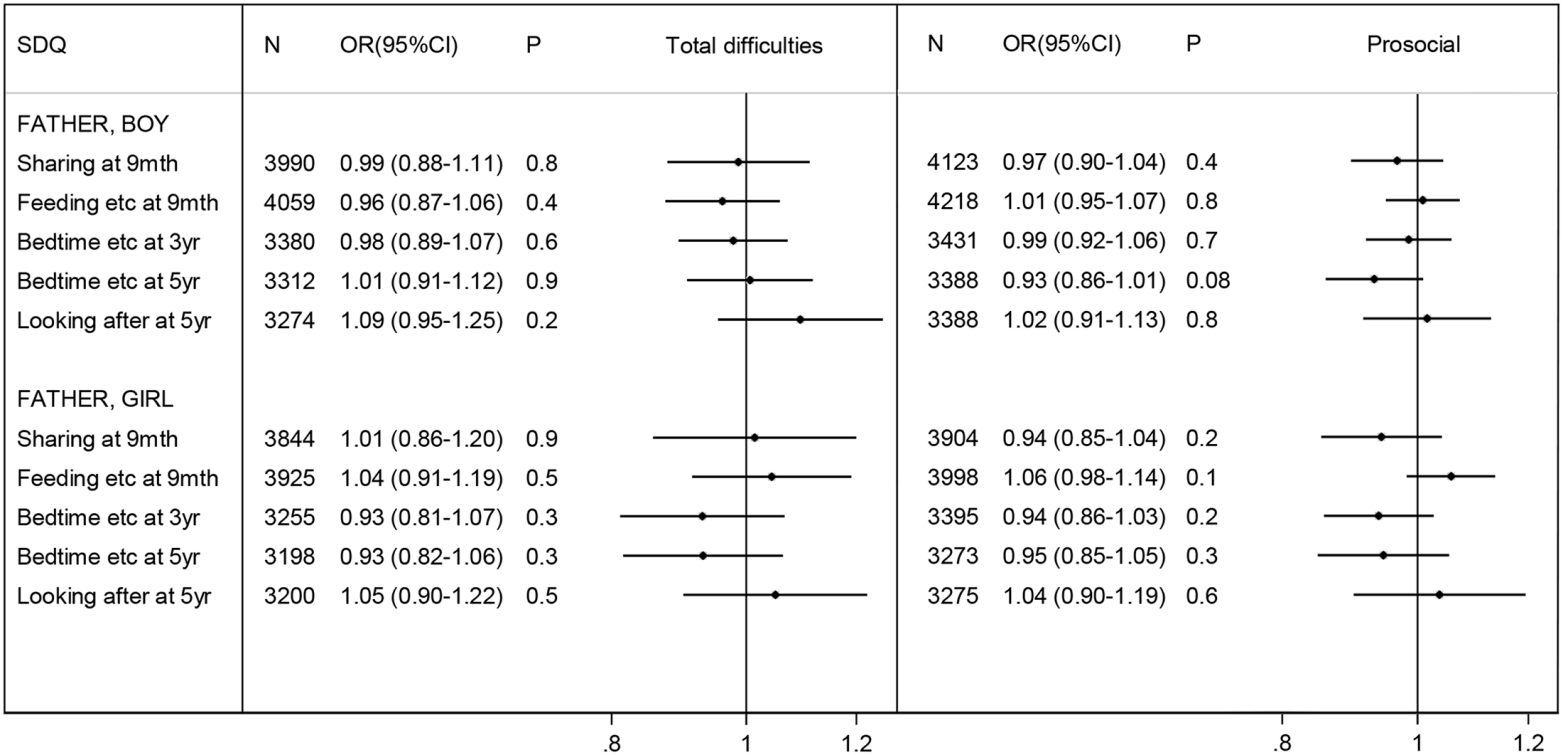
Adjusted odds ratio for child behaviour problems per quintile^a^ of involvement: father-only measures. See Table 3 for adjustment factors. Involvement at 9 months, 3 years, 5 years (see Table 2 for details). Behaviour problems respectively at 3, 5, 7 years, i.e. SDQ score in the top decile for the total difficulties scale, bottom decile for the prosocial scale. ^**a**^ per quartile for ‘Sharing at 9mth’.

Except for ‘looking after alone’ at age 5 years, as previously mentioned, the distributions of these measures did not vary significantly between boys and girls (Fig 1). Using the SDQ total difficulties scale, before adjustment for confounders, bedtime/reading/play at 3 years and bedtime/reading at 5 years were associated with lower risks of behaviour problems in girls (Table 5); after adjustment, there were no statistically significant associations for either boys or girls (Fig 3). Using the SDQ prosocial scale, before adjustment for confounders, bedtime/reading at 5 years was associated with lower risk of behaviour problems in boys (Table 5); after adjustment there were no significant associations (Fig 3).

**Table 5:** Crude odds ratio for child behaviour problems per quintile^a^ of involvement: father-only measures.

SDQ	Total difficulties			Prosocial		
Involvement	N	OR (95% CI)	P	N	OR (95% CI)	P
**FATHER, BOY**						
Sharing care at 9mth	4076	1.05 (0.94–1.18)	0.3	4124	0.95 (0.88–1.02)	0.2
Feeding etc. at 9mth	4167	0.99 (0.91–1.09)	0.9	4219	1.00 (0.94–1.07)	0.9
Bedtime etc. at 3yr	3527	0.92 (0.85–1.01)	0.07	3540	0.96 (0.89–1.02)	0.2
Bedtime etc. at 5yr	3374	0.93 (0.84–1.02)	0.1	3389	0.92 (0.85–0.99)	0.04
Looking after at 5yr	3374	1.12 (0.97–1.28)	0.1	3389	1.02 (0.91–1.13)	0.8
**FATHER, GIRL**						
Sharing care at 9mth	3909	1.13 (0.97–1.31)	0.1	3961	0.93 (0.84–1.02)	0.1
Feeding etc. at 9mth	4007	1.06 (0.93–1.21)	0.4	4060	1.05 (0.98–1.13)	0.2
Bedtime etc. at 3yr	3376	0.84 (0.74–0.96)	0.009	3396	0.93 (0.85–1.02)	0.1
Bedtime etc. at 5yr	3291	0.82 (0.73–0.92)	0.001	3305	0.92 (0.83–1.02)	0.1
Looking after at 5yr	3293	1.08 (0.93–1.24)	0.3	3307	1.04 (0.91–1.19)	0.6

Involvement at 9 months, 3 years, 5 years (see Table 2 for details). Behaviour problems respectively at 3, 5, 7 years, i.e. SDQ score in the top decile for the total difficulties scale, bottom decile for the prosocial scale

^**a**^ per quartile for ‘Sharing care at 9mth’.

### Composite involvement measures that were available for both parents

**Fig 4:**
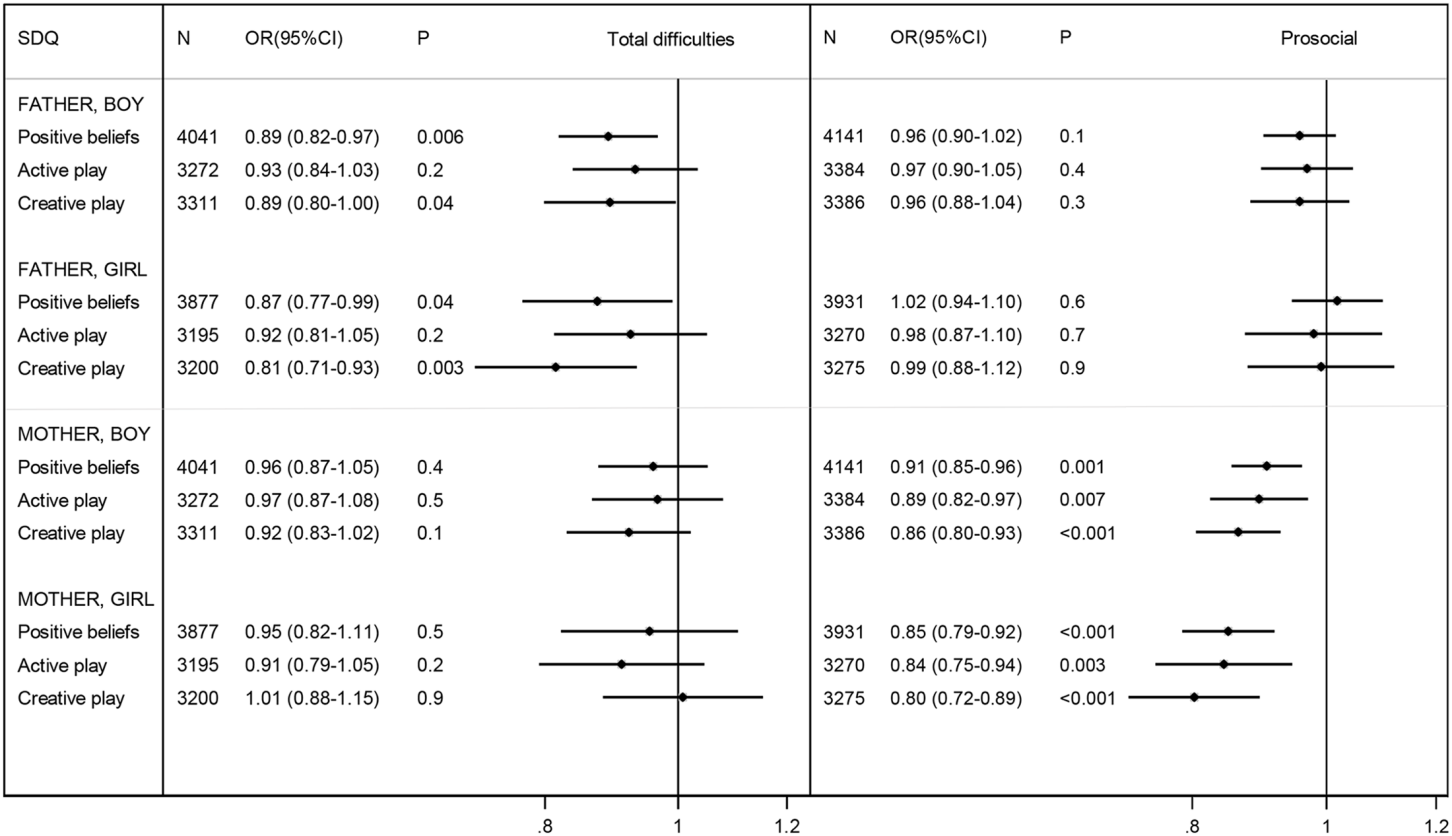
Adjusted odds ratio for child behaviour problems per quintile of involvement: both-parent measures. Positive parenting beliefs at 9 months, active/creative play at 5 years (see Table 2 for details). Behaviour problems respectively at 5, 7 years: SDQ score in the top decile for the total difficulties scale, or bottom decile for the prosocial scale. See Table 3 for adjustment factors.

Father involvement in active play was greater for boys than girls (p<0.001), and mother involvement in creative play was greater for girls than boys (p<0.001) (Fig 2). Fathers’ and mothers’ parenting beliefs, mothers’ active play, and fathers’ creative play did not vary with the sex of the child. Using the SDQ total difficulties scale, nearly all these involvement measures were associated with lower risks of behaviour problems in boys and/or girls before adjustment for confounders (Table 6). After adjustment, which included control for equivalent co-parent involvement, two measures of father involvement (positive parenting beliefs at 9 months and creative play at 5 years) remained significant for both boys and girls, the odds ratios ranging between 0.81 and 0.89 per quintile (Fig 4); there were no significant associations for mothers. Using the SDQ prosocial scale, before adjustment for confounders, involvement was associated with lower risks of behaviour problems in both boys and girls for mothers, but only in boys for fathers (Table 6). After adjustment, all three measures of mother involvement remained significant for boys (odds ratios ranging between 0.86 and 0.91 per quintile) and girls (odds ratios ranging between 0.80 and 0.85 per quintile) (Fig 4); there were no significant associations for fathers.

**Table 6:** Crude odds ratio for child behaviour problems per quintile of involvement: both-parent measures.

SDQ	Total difficulties			Prosocial		
Involvement	N	OR (95% CI)	P	N	OR (95% CI)	P
**FATHER, BOY**						
Positive beliefs at 9mth	4105	0.83 (0.76–0.89)	<0.001	4155	0.93 (0.87–0.98)	0.01
Active play at 5yr	3373	0.89 (0.81–0.99)	0.03	3387	0.93 (0.86–1.00)	0.06
Creative play at 5yr	3373	0.89 (0.80–1.00)	0.04	3388	0.92 (0.85–1.00)	0.04
**FATHER, GIRL**						
Positive beliefs at 9mth	3948	0.80 (0.71–0.89)	<0.001	3999	0.98 (0.91–1.05)	0.6
Active play at 5yr	3292	0.86 (0.76–0.97)	0.01	3306	0.92 (0.82–1.03)	0.2
Creative play at 5yr	3293	0.79 (0.70–0.90)	<0.001	3307	0.91 (0.80–1.02)	0.1
**MOTHER, BOY**						
Positive beliefs at 9mth	4147	0.84 (0.77–0.92)	<0.001	4197	0.87 (0.83–0.92)	<0.001
Active play at 5yr	3373	0.91 (0.82–1.00)	0.06	3388	0.88 (0.81–0.95)	0.001
Creative play at 5yr	3374	0.92 (0.83–1.01)	0.08	3389	0.85 (0.79–0.91)	<0.001
**MOTHER, GIRL**						
Positive beliefs at 9mth	3986	0.80 (0.70–0.90)	<0.001	4040	0.84 (0.79–0.91)	<0.001
Active play at 5yr	3292	0.82 (0.72–0.93)	0.002	3306	0.81 (0.73–0.91)	<0.001
Creative play at 5yr	3296	0.94 (0.84–1.05)	0.3	3310	0.78 (0.71–0.86)	<0.001

Positive parenting beliefs at 9 months, active/creative play at 5 years (see Table 2 for details). Behaviour problems respectively at 5, 7 years, i.e. SDQ score in the top decile for the total difficulties scale, bottom decile for the prosocial scale.

## Discussion

### Summary of results

In this large prospective study of UK two-parent families, two measures of father involvement (positive parenting beliefs at age 9 months and frequency of creative play at age 5 years) were associated with lower risk of subsequent behaviour problems, assessed using the SDQ total difficulties scale, in both boys and girls. Other composite measures of caring activity by the father at 9 months, 3 years or 5 years were not associated with child behavioural outcomes.

Where available, equivalent measures of mother involvement (positive parenting beliefs at 9 months and engagement in both active and creative play at 5 years) were associated with lower risk of subsequent behaviour problems, assessed using the SDQ prosocial scale, in both boys and girls.

### Comparison with other studies

Our results suggest that the father’s quality of parenting, rather than frequency or share of routine care, is associated with lower risk of child behaviour problems. This finding is consistent with a recent analysis of data from a large Australian cohort (the Longitudinal Study of Australian Children, LSAC), which reported that child behaviour was positively associated with quality of parenting by the father (warmth, self-efficacy and good co-parental relationship), but not with father’s contact time, after adjusting for mother’s parenting and many other potential confounders [37].

In adjusted models of father involvement, using the SDQ total difficulties score, we found no statistically significant association of behavioural outcomes with frequency or share of routine care at 9 months, 3 years or 5 years. Previous MCS studies have related broad composite measures of father engagement in caring activities to subsequent child behavioural outcomes using subscales of total difficulties (emotion, conduct, attention or peer problems) [38–40]: most effects were very small and not statistically significant, but (among the large number of analyses performed), inverse associations were reported for (a) engagement at 9 months with emotional problems at 3 years [38], (b) engagement at 3 years with attention problems at 5 years [39] and (c) engagement at 5 years with peer problems at 7 years [40]. To our knowledge, no previous MCS study has assessed the measures for which we found significant associations, i.e. positive parenting beliefs at 9 months, and creative play (separately from other activities) at 5 years.

Other evidence for effects of father involvement on child behaviour is limited. A systematic review of publications up to September 2007 concluded that ‘there is evidence to support the positive influence of father engagement on offspring social, behavioural and psychological outcomes’, but noted that almost all the studies were subject to methodological limitations [14]. Most of the publications examined older children or teenagers, and all the UK evidence came from a cohort of children born in 1958. More recently, a report described parallel analyses of cohort studies from the UK (i.e. the MCS), Australia (i.e. the LSAC), Denmark, and the USA [39]. In the MCS analysis (as previously mentioned) there was a tendency for children with highly-engaged fathers to have lower risk of attention problems. No associations were observed in the other cohorts, and the authors of the report commented that during the early 2000s father involvement might have been more closely linked to socioeconomic status in the UK than in the other three countries.

We found that paternal and maternal parenting characteristics were associated with different types of child behaviour. This is consistent with observational studies that have reported associations of maternal parenting quality with empathic/prosocial behaviour [41, 42], and paternal parenting quality with successful coping with negative emotions and lower risk of externalising problems [41, 43] in young children.

### Potential mechanisms

There is evidence that more ‘authoritative’ (demanding and responsive) parenting is associated with better behavioural outcomes [44, 45]. ‘Demanding’ means that the parent sets clear rules and asserts them firmly but not aggressively; ‘responsive’ means that the parent observes and respects the child’s thoughts and emotions, and responds with warmth and sensitivity. In our analysis, a belief in the importance of regularity, stimulation, cuddling, talking and father involvement at 9 months seems analogous to a belief in the efficacy of authoritative parenting. Assuming that this belief is put into practice, the inverse association with subsequent child behaviour problems might be expected.

At age 9 months, feeding may be less important as a mechanism for building the relationship between baby and carer than it is in the first few months of life [46]. Nappy-changing and ‘looking after’ do not necessarily entail the carer’s full attention. Moreover, all these essential childcare tasks would be carried out by someone else (e.g. the mother) if not by the father. Hence, their lack of clear association with behavioural outcomes in studies of father involvement, including ours, is perhaps unsurprising. Other caring tasks (bedtime, reading, play) have a potential for more complex interaction, and some (reading, play) do not have a fixed total frequency, so that both parents can contribute independently. Creative play (art, music, story-telling, making things) may entail particularly focussed and responsive parental attention. This may explain the inverse association between creative play and behaviour problems seen in our study.

Some types of parent involvement may be partly determined by the behaviour of the child. For example, studies using path analysis have found weak bi-directional associations of parent involvement with child behaviour, and a tendency for children with hyperactivity or conduct problems to have less-involved fathers at subsequent ages [40, 47]. In our study, persistent child behaviour problems may have deterred fathers from engaging in creative play at age 5 years. Reverse causation seems unlikely to explain the observed association of positive parenting beliefs at 9 months with child behaviour at 3 years, however.

Where equivalent measures of mother involvement were available, we found associations with risk of behaviour problems using the SDQ prosocial scale (but not the SDQ total difficulties scale) for mothers, and using the total difficulties (but not prosocial) scale for fathers. The results for mother involvement should be interpreted with caution because both exposure and outcome were assessed by the mother, albeit at quite widely spaced time points. Nevertheless, it is possible that the influence of parental behaviour modelling on the child’s social skills may have been stronger for mothers than fathers, because, in this sample, the mothers did the majority of day-to-day care.

We found no evidence that associations of father involvement with child behaviour were stronger for boys than girls. However, we noted that, after infancy, father engagement in some of the individual recorded activities was slightly greater for boys than girls, perhaps reflecting shared interests and/or greater confidence with boys; both parents tended to be more involved in physically active play with boys, and musical activities with girls, perhaps in response to underlying biological or psychosocial differences in the children [48].

### Strengths and limitations

This study was a prospective analysis of the most recent available nationally-representative cohort data from the UK. Population sizes were large enough to allow separate models for boys and girls. Composite measures of father involvement were derived by exploratory factor analysis, and equivalent measures of mother involvement were examined where possible. Two separate SDQ scales were used to measure different aspects of child behaviour. All statistically significant odds ratios were expressed in standard units (quintiles of the analysis population) for internal comparability, and controlled for co-parent involvement, child temperament, and other confounders.

Care is needed when extrapolating the results to current UK families, in view of continuing change in parental roles. By restricting the study to English-speaking families with co-resident birth parents, we were able to minimise missing data, and focus on quality and quantity of father involvement rather than the impact of having a non-resident father, but the results may not be generalizable to other cultures or family structures. When interpreting the results, it should be noted that the source data were parent-reported, not observational. Observer bias is unlikely to have influenced the main results for father involvement, as exposure and outcome were assessed by different people (father and mother respectively), but this was not the case for mother involvement.

### Conclusions

Among two-parent families in the UK, positive parenting beliefs and engagement in creative play by the father were associated with a lower risk of subsequent behaviour problems for both boys and girls. There was no strong evidence of association with frequency or share of other parental caring activities by the father at 9 months, 3 years or 5 years. These findings suggest that quality of parenting, rather than the division of routine care between parents, is important for child behavioural outcomes. Interventions should aim to improve support and training for new fathers, with an emphasis on parenting quality.
